# Identification of *Elizabethkingia* species by MALDI-TOF MS proteotyping

**DOI:** 10.1128/spectrum.02454-24

**Published:** 2025-02-06

**Authors:** Satomi Takei, Kanae Teramoto, Yuji Sekiguchi, Takashi Miida, Teruo Kirikae, Tatsuya Tada, Yoko Tabe

**Affiliations:** 1 Department of Clinical Laboratory Medicine, Juntendo University Graduate School of Medicine, Tokyo, Japan; 2 Department of MALDI-TOF MS Practical Application Research, Juntendo University Graduate School of Medicine, Tokyo, Japan; 3 Analytical and Measurement Instruments Division, Shimadzu Corporation, Kyoto, Japan; 4 Biomedical Research Institute, National Institute of Advanced Industrial Science and Technology (AIST), Tsukuba, Ibaraki, Japan; 5 Department of Clinical Medical Technology, Faculty of Medical Science, Juntendo University, Urayasu, Japan; 6 Department of Microbiome Research, Juntendo University Graduate School of Medicine, Tokyo, Japan; 7 Department of Microbiology, Juntendo University Graduate School of Medicine, Tokyo, Japan; Instituto de Investigacion Sanitaria Gregorio Maranon, Madrid, Spain

**Keywords:** *Elizabethkingia* species, MALDI-TOF MS, whole-genome, ribosomal protein, YtxH domain-containing proteins

## Abstract

*Elizabethkingia* species, isolated from clinical and environmental samples, are emerging opportunistic bacterial pathogens with a high mortality rate in clinical settings worldwide. Taxonomically, *Elizabethkingia* comprises seven species: *E. anophelis*, *E. argenteiflava*, *E. bruuniana*, *E. meningoseptica*, *E. miricola*, *E. ursingii*, and *E. occulta*. In this study, we identified useful biomarker proteins, including ribosomal L29, L30, S21, and the YtxH domain-containing proteins, for distinguishing *Elizabethkingia* species using matrix-assisted laser desorption/ionization time-of-flight mass spectrometry (MALDI-TOF MS) profiles. Evaluation of 29 clinical and environmental *Elizabethkingia* strains revealed that these species could be separated by MALDI-TOF MS profiles into six groups*—E. anophelis*, *E. argenteiflava*, *E. bruuniana/E. miricola*, *E. meningoseptica*, *E. ursingii*, and *E. occulta*—based on the four biomarker protein peaks. This study demonstrates the potential of routine MALDI-TOF MS -based examination methods for the early detection of *Elizabethkingia* species in clinical laboratories.

**IMPORTANCE:**

*Elizabethkingia* species are groups of emerging opportunistic bacterial pathogens with a high mortality rate, causing healthcare-associated outbreaks worldwide. Rapid identification of *Elizabethkingia* species is important becausethese species show intrinsically carbapenem resistance and there are few data for using appropriate antibiotics. Until now, only whole-genome sequencing could accurately identify the seven *Elizabethkingia* species. Therefore, establishing rapid and accurate identification methods for *Elizabethkingia* species in clinical laboratories is vital. In this study, we developed new methods for identifying *Elizabethkingia* species using four biomarker protein peaks-ribosomal L29, L30, S21, and the YtxH domain-containing proteins by matrix-assisted laser desorption/ionization time-of-flight mass spectrometry (MALDI-TOF MS) proteotyping. This study demonstrates the potential of routine MALDI-TOF MS -based laboratory examination for the early identification of *Elizabethkingia* species.

## INTRODUCTION

Members of the genus *Elizabethkingia* are known to be Gram-negative, aerobic, non-fermenting, nonmotile, and rod-shaped bacteria (1) . A bacterium of the genus was first found in 1959 by Elizabeth O. King and was described as “*Flavobacterium meningosepticum*” (2). Now, the relevant taxa were transferred to *Elizabethkingia*, in which seven species are validly published as *E. anophelis*, *E. argenteiflava*, *E. bruuniana*, *E. meningoseptica*, *E. miricola*, *E. ursingii*, and *E. occulta* (3). *E. anophelis* was first isolated from the midgut of *Anopheles gambiae* mosquitos in Africa in 2011 (4); *E. argenteiflava* from soybean in South Korea in 2015 (5); *E. bruuniana* from human blood in the United Kingdom in 1975 (6); *E. meningoseptica* from cerebrospinal fluid, blood, and throat of infants in the United States in 1959 (2); *E. miricola* from condensation water on the Mir space station of Russia in 1997 (7); *E. ursingii* from soil in Denmark in 1964 (6); and *E. occulta* from human sputum in Australia in 1977 (6).

Clinical strains of the known *Elizabethkingia* species, which are isolated from respiratory, blood, and urine samples often cause severe infections in neonatal or immunocompromised patients, with high mortality rates of 25–52% (8, 9) and have caused hospital outbreaks worldwide. *E. anophelis* infections have caused outbreaks in 10 countries (10), including Argentina, Australia, China, France, Hong Kong, India, Singapore, South Korea, Taiwan, and the United States. *E. meningoseptica* infections have caused outbreaks in children in 10 countries (11), including Brazil, Denmark, India, Israel, Mauritius, Norway, Taiwan, Turkey, the United Kingdom, and the United States. *E. miricola* infections have caused an outbreak in Spain (12). Other known *Elizabethkingia* species, except for *E. argenteiflava*, have also been isolated from human specimens (5).

The majority of known clinical strains of *Elizabethkingia* species are resistant to all β-lactams due to intrinsic metallo-β-lactamases and extended-spectrum β-lactamase, to aminoglycosides by aminoglycoside-6-adenyl transferases, and to colistin by intrinsic phosphoethanolamine transferase (13, 14). The susceptibility patterns to fluoroquinolones and trimethoprim/sulfamethoxazole vary among *Elizabethkingia* strains (14). Distinguishing the known species of the genus *Elizabethkingia* by susceptibility patterns is challenging and no first-line antibiotics are currently available.

In clinical laboratories, *Elizabethkingia* species are identified using biochemical methods such as API/ID32 Phenotyping Kits (bioMérieux, Marcy l’Etoile, France), Phoenix 100 ID/AST Automated Microbiology System (Becton Dickinson Co., Sparks, MD, USA), and Vitek 2 Automated Identification System (bioMérieux); mass spectrometry methods such as Vitek MS (bioMérieux) and Bruker Biotyper MS (Bruker Daltonics GmbH, Bremen, Germany); and by sequencing of 16S rRNA gene or whole-genomes (3). While whole-genome sequencing (WGS) can correctly identify *Elizabethkingia* at the species level, it is not routinely used due to its high cost and time-consuming procedures.

Correct identification of *Elizabethkingia* at the species level is often vital to distinguish and to prevent hospital outbreaks. Up to now, the epidemiological information on *Elizabethkingia* species is not sufficient; therefore, it is important to accumulate surveillance data from clinical laboratories. In this study, we employed the matrix-assisted laser desorption/ionization time-of-flight mass spectrometry (MALDI-TOF MS)-based microbial identification method and explored biomarker mass peaks for distinguishing the species of *Elizabethkingia*. Detected biomarker mass peaks were annotated based on the genomically predicted protein mass database (GPMsDB) (15) , in which theoretical protein masses were predicted based on genome sequences, including those of the genus *Elizabethkingia*.

## MATERIALS AND METHODS

### Bacterial strains

The type strains of the known species of *Elizabethkingia* were used to obtain MALDI-TOF MS mass spectra. The type strain of *E. meningoseptica* NBRC 12535^T^ was obtained from Biological Resource Center, National Institute of Technology and Evaluation (Tokyo, Japan); *E. anophelis* NCTC 13869^T^ from National Collection of Type Cultures (Salisbury, the United Kingdom); *E. miricola* JCM 11413^T^ and *E. argenteiflava* JCM 32097^T^ from RIKEN BRC (Tsukuba, Ibaraki, Japan); and *E. bruuniana* CIP 111191^T^, *E. ursingii* CIP 111192^T^, and *E. occulta* CIP 111193^T^ from Collection de l'Institut Pasteur (Paris, France). In addition, *E. meningoseptica* CCUG 69507 and CCUG 69515; *E. miricola* CCUG 69494 and CCUG 69519; *E. bruuniana* CCUG 69504, CCUG 69513, and CCUG 69522; *E. ursingii* CCUG 69498 and CCUG 69517; and *E. occulta* CCUG 69497 were obtained as additional reference strains from Culture Collection University of Göteborg (CCUG) (Göteborg, Sweden). Eight clinical strains of *Elizabethkingia* were obtained from eight patients treated at Juntendo University Hospital in Japan between August 2015 and January 2024. Three strains of *Elizabethkingia* were obtained from mosquitos in Japan from August to September 2023, and one strain from a mosquito in Thailand in July 2023 (Table S1). These strains were cultured on 5% sheep blood agar (Becton, Dickinson-Diagnostic Systems) under aerobic conditions at 35°C.

### WGS

Genomic DNA of the eight clinical and four environmental strains of *Elizabethkingia* species were extracted using DNeasy blood and tissue kits (Qiagen, Tokyo, Japan). DNA libraries were prepared using Nextera XT DNA Library Prep Kit (Illumina, San Diego, CA, USA). The genomes were sequenced on the Illumina MiSeq platform using v3 chemistry (600 cycles) or the Illumina MiniSeq platform (300 cycles), and the summary of the assembly is shown in Table S1. Raw reads for each strain were trimmed and assembled using CLC Genomic Workbench version 10.0.1 (CLC bio, Aarhus, Denmark). Genome relatedness of the relevant strains was estimated using an average nucleotide identity (ANI) calculator (16), a Type (Strain) Genome Sever (https://tygs.dsmz.de), and 16S rRNA gene sequences. ANI values and 16S rRNA gene sequence identities were calculated using reference genomes of *E. anophelis* (NCTC 13869^T^; genome accession number GCF_002023665), *E. argenteiflava* (JCM 32097^T^; GCF_009904105), *E. bruuniana* (CIP 111191^T^; GCF_002024805), *E. meningoseptica* (NBRC 12535^T^; GCF_900475375), *E. miricola* (JCM 11413^T^;GCF_008124555), *E. ursingii* (CIP 111192^T^; GCF_001521765), and *E. occulta* (CIP 111193^T^; GCF_002023715).

### Phylogenetic analysis

Genome completeness and contamination were assessed using CheckM2 v1.0.1 with lineage_wf and default settings (17). A phylogenetic tree was constructed using the kSNP4 software based on the pangenome SNPs (https://sourceforge.net/projects/ksnp/) (18), and visualized using iTol ver.6 (https://itol.embl.de/). The strains of *E. anophelis* (NCTC 13869^T^; GCF_002023665), *E. argenteiflava* (JCM 32097^T^; GCF_009904105), *E. bruuniana* (CIP 111191^T^; GCF_002024805, CCUG 69504; GCF_002023775, and CCUG 69513; GCF_002023765), *E. meningoseptica* (NBRC 12535^T^; GCF_900475375, CCUG 69507; GCF_002022105 and CCUG 69515; GCF_002023305), *E. miricola* (JCM 11413^T^; GCF_008124555, CCUG 69494; GCF_001521745 and CCUG 69519; GCF_002023515), *E. ursingii* (CIP 111192^T^; GCF_001521765, CCUG 69498; GCF_002023365 and CCUG 69517; GCF_002023405), and *E. occulta* (CIP 111193^T^; GCF_002023715 and CCUG 69497; GCF_002023385) were used as reference strains.

### Calculation of the theoretical mass of *Elizabethkingia* species for MALDI-TOF MS proteotyping

Theoretical masses of proteins encoded in the genomes of *Elizabethkingia* were calculated for the following genomes as part of the development of a GPMsDB: *E. anophelis* (NCTC 13869^T^; genome accession number GCF_002023665), *E. argenteiflava* (JCM 32097^T^; GCF_009904105), *E. bruuniana* (CIP 111191^T^; GCF_002024805), *E. meningoseptica* (NBRC 12535^T^; GCF_900475375), *E. miricola* (JCM 11413^T^; GCF_008124555), *E. ursingii* (CIP 111192^T^; GCF_001521765), and *E. occulta* (CIP 111193^T^; GCF_002023715) (15). The genome sequences were obtained from the NCBI database (https://www.ncbi.nlm.nih.gov/). Gene prediction from the genomes obtained in this study was performed using GPMsDB-dbtk v1.0.1 (https://github.com/ysekig/GPMsDB-dbtk) (15).

### Bacterial sample preparation for MALDI-TOF MS

Alpha-cyano-4-hydroxycinnamic acid (CHCA) was used as a matrix. To prepare this matrix solution, 10 mg of 4-CHCA was dissolved in 1 mL of solvent consisting of 1% (vol/vol) trifluoroacetic acid, 35% (vol/vol) ethanol, 15% (vol/vol) acetonitrile, and milliQ water. A full loop of bacterial cells was dispersed in 200 µL of distilled water in a microtube and mixed with 800 µL of ethanol. The suspensions were briefly vortexed and centrifuged at 15,000 × *g* for 2 min. The pellets were then dried for 5 min. The pellets were suspended in 50 µL of 70% formic acid, vortexed, suspended in 50 µL of acetonitrile, and centrifuged at 15,000 × *g* for 2 min. Supernatants were analyzed by MALDI-TOF MS according to the manufacturer’s instructions.

### MALDI-TOF MS measurement

MALDI-TOF MS measurements were performed in positive linear mode using MALDI-8020 RUO (Shimadzu Corporation, Japan) and Microflex LT/SH (Bruker Daltonics, Germany) equipped with a 200 Hz Nd:YAG laser (355 nm) and 60 Hz nitrogen laser (337 nm), respectively. Before sample analysis, the MALDI-TOF MS instrument was mass-calibrated externally using six peaks with *m/z* 4,365.4, 5,381.4, 6,411.6, 7,274.0, 8,369.8, and 10,300.1 from *Escherichia coli* DH5α. Five individual mass spectra were acquired for each bacterial extract in the range of *m/z* 2,000–20,000. Peak assignment was carried out using eMSTAT Solution software (Shimadzu Corp.). Species identification was performed by MBT Compass 4.1 with Microflex LT/SH (Bruker Daltonics).

### Cluster analysis

For biomarker validation, 29 *Elizabethkingia* strains, including the seven-type strains were analyzed by MALDI-TOF MS. Five mass spectra were acquired for each strain, and peak lists were extracted from those mass spectra. Biomarker analysis was performed using the binary biomarker matching table, which scored peaks as either 1 or 0, and a dendrogram was constructed using the unweighted pair group method with arithmetic mean using SRplot (19).

## RESULTS

### Identification of *Elizabethkingia* strains from clinical and environmental samples by WGS

**Fig 1:**
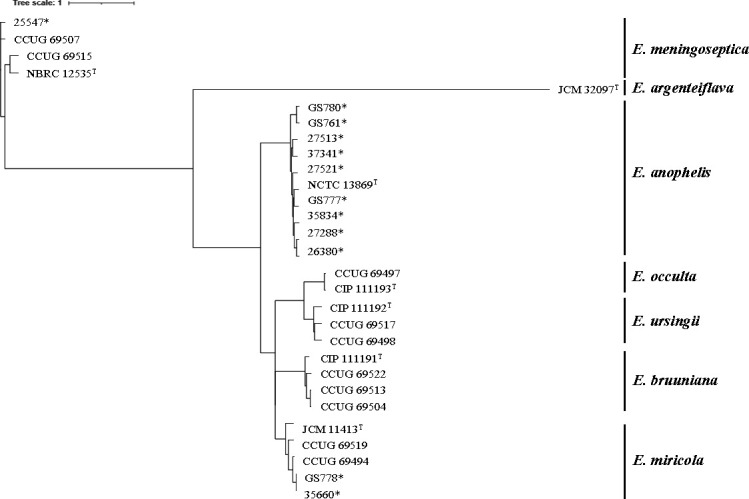
Phylogenetic tree of 12 clinical or environmental strains, and 17 type and reference strains of *Elizabethkingia* species, including *E. anophelis*, *E. argenteiflava, E. bruuniana, E. meningoseptica*, *E. miricola*, *E. ursingii*, and *E. occulta*. Phylogenetic trees were constructed using kSNP4 software based on the pangenome SNPs (https://sourceforge.net/projects/ksnp/) (18) and visualized using iTol ver.6 (https://itol.embl.de/). The strains marked with asterisks indicate the 12 tested clinical or environmental strains (test strains).

Phylogenetic analysis based on WGS showed that the 29 strains examined in this study (12 test strains and 17 reference strains) belonged to distinct groups representing species-level clades of *Elizabethkingia*, namely *E. anophelis*, *E. argenteiflava*, *E. bruuniana*, *E. meningoseptica*, *E. miricola*, *E. ursingii*, and *E. occulta* (Fig. 1). Of the 12 test strains, 9 were clustered within the *E. anophelis* group, 2 were in the *E. miricola* group, and 1 was in *E. meningoseptica* (Table S1).

### Comparison of *Elizabethkingia* strains by WGS and 16S rRNA gene sequences

The ANI values among *Elizabethkingia*-type strains were obtained using WGS data. Seven-type strains representing *Elizabethkingia* species were clearly distinguished by WGS using the ANI calculator with a 95% cutoff value. However, it seems difficult to distinguish *E. bruuniana*, *E. miricola*, *E. ursingii*, and *E. occulta* strains based on 16S rRNA gene sequencing because of 98.80–99.60% identities among these species (Table S2).

### Identification of *Elizabethkingia* species by MALDI-TOF MS using type strains

**Fig 2:**
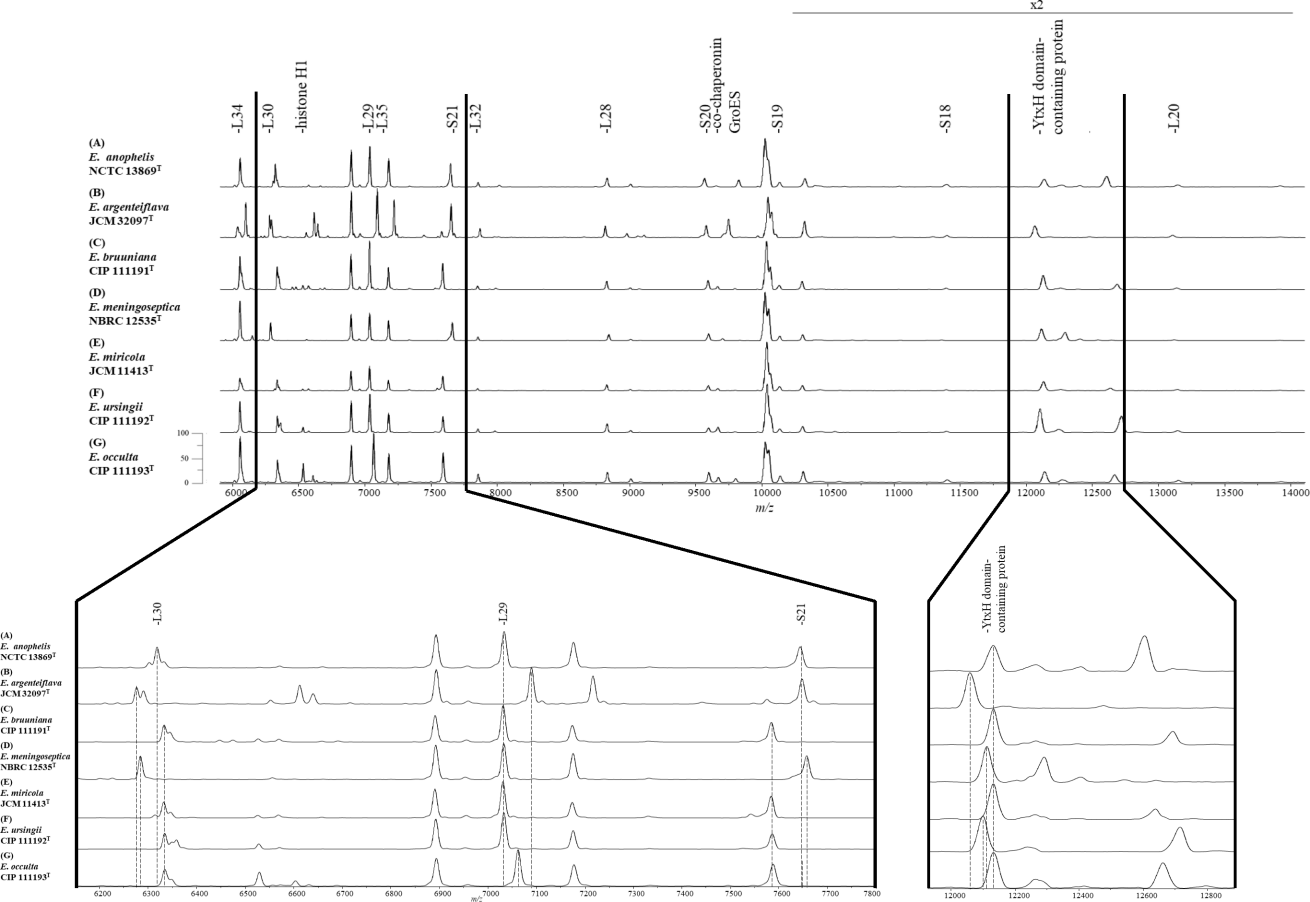
Representative mass spectra of *Elizabethkingia* species, including *E. anophelis* NCTC 13869^T^ (**A**), *E. argenteiflava* JCM 32097^T^ (**B**)*, E. bruuniana* CIP 111191^T^ (**C**)*, E. meningoseptica* NBRC 12535^T^ (**D**), *E. miricola* JCM 11413^T^ (**E**), *E. ursingii* CIP 111192^T^ (**F**), and *E. occulta* 111193^T^ (**G**). Upper figure indicates mass spectra from *m/z* 6,000 to 14,000. The annotated peaks indicate the assigned peaks based on calculated masses within the tolerance at 500 ppm. Amplification of the *m/z* 6,200–7,800 section of mass spectra results (lower figure) reveals variations of *m/z* values for peaks ribosomal L29, L30, and S21 and the *m/z* 12,000–12,800 section of mass spectra results reveals variations of *m/z* values for peaks YtxH domain-containing proteins.

The mass peaks identified using MALDI-TOF MS with the seven-type strains representing all the known *Elizabethkingia* species are shown in Fig. 2. The calculated and observed masses are summarized in Table 1. In the MALDI-TOF MS profiles, 14 major mass peaks were detected and successfully annotated with predicted protein names. Of these 14 annotated peaks, 11 were predicted as ribosomal subunit proteins, and the remaining three were annotated as histone H1, co-chaperonin GroES, and YtxH domain-containing proteins. As shown in Table 1, compared with the theoretical mass peaks of *E. anophelis* NCTC 13869^T^, 12, 5, 9, 5, 6, and 6 peaks were different from the theoretical mass peaks of *E. argenteiflava* JCM 32097^T^*, E. bruuniana* CIP 111191^T^, *E. meningoseptica* NBRC 12535^T^, *E. miricola* JCM 11413^T^, *E. ursingii* CIP 111192^T^, and *E. occulta* CIP 111193^T^, respectively.

**TABLE 1:** Annotated peaks of type strains of *Elizabethkingia* species^*a*^

Biomarker proteins	Species																				
	*Elizabethkingia anophelis* NCTC 13869^T^			*Elizabethkingia argenteiflava* JCM 32097^T^			*Elizabethkingia bruuniana* CIP 111191^T^			*Elizabethkingia meningoseptica* NBRC 12535^T^			*Elizabethkingia miricola* JCM 11413^T^			*Elizabethkingia ursingii* CIP 111192^T^			*Elizabethkingia occulta* CIP 111193^T^		
	Calculated masses (*m/z*)	Average	SE	Calculated masses (*m/z*)	Average	SE	Calculated masses (*m/z*)	Average	SE	Calculated masses (*m/z*)	Average	SE	Calculated masses (*m/z*)	Average	SE	Calculated masses (*m/z*)	Average	SE	Calculated masses (*m/z*)	Average	SE
L34	6,052.1	6,052.5	0.23	NA	NA	NA	6,052.1	6,051.9	0.58	6,052.1	6,053.1	0.20	6,052.1	6,052.8	0.23	6,052.1	6,051.8	0.26	6,052.1	6,052.2	0.30
L30	6,317.5	6,318.2	0.19	6,274.4	6,275.3	0.05	6,333.5	6,333.4	0.51	6,283.5	6,284.1	0.31	6,333.5	6,334.0	0.20	6,333.5	6,334.6	0.32	6,333.5	6,333.6	0.21
Histone H1	6,527.5	6,529.9	0.50	6,610.6	6,611.3	0.06	6,527.5	6527.3	0.49	6,598.6	6,600.2	0.29	6,527.5	6,527.4	0.25	6,527.5	6,528.3	0.28	6,527.5	6,527.9	0.21
L29	7,032.1	7,032.6	0.12	7088.2	7088.5	0.03	7,032.1	7,032.1	0.40	7,032.1	7,032.5	0.15	7,032.1	7,032.4	0.13	7,032.1	7,032.8	0.18	7,060.1	7,060.3	0.08
L35	7,174.6	7,175.3	0.12	7,214.7	7,215.3	0.16	7,174.6	7,174.8	0.38	7,174.6	7,175.1	0.12	7,174.6	7,175.1	0.15	7,174.6	7,175.3	0.13	7,174.6	7,175.2	0.11
S21	7,643.0	7,643.0	0.08	7,647.0	7,647.0	0.05	7,585.9	7,585.7	0.32	7,657.0	7,657.0	0.08	7,585.9	6,068.5	0.07	7,585.9	7,586.0	0.06	7,585.9	7,586.0	0.03
L32	7,850.8	7,851.1	0.09	7,864.9	7,864.9	0.09	7,849.8	7,849.7	0.34	7,850.8	7,850.9	0.22	7,849.8	7,849.9	0.13	7,849.8	7,849.7	0.09	7,849.8	7,849.9	0.10
L28	8,826.3	8,825.7	0.07	8,812.3	8,811.6	0.14	8,826.3	8,825.7	0.25	8,840.3	8,839.6	0.11	8,826.3	8,825.3	0.16	8,826.3	8,825.4	0.15	8,826.3	8,825.7	0.11
S20	9,564.3	9,562.9	0.25	9,576.3	9,574.8	0.06	9,594.3	9,593.1	0.32	9,594.3	9,593.2	0.27	9,594.3	9,593.4	0.32	9,594.3	9,593.0	0.28	9,594.3	9,593.2	0.22
Co-chaperonin GroES	9,822.5	9,821.2	0.25	9,744.2	9,743.4	0.17	9,665.2	9,664.8	0.37	9,699.2	9,698.3	0.36	9,665.2	9,664.1	0.31	9,665.2	9,664.1	0.32	9,665.2	9,664.7	0.17
S19	10,129.7	10,130.0	0.27	10,102.7	NA	NA	10,129.7	10,130.4	0.83	10,129.7	10,130.3	0.22	10,129.7	10,129.7	0.26	10,129.7	10,129.9	0.66	10,129.7	10,131.0	0.46
S18	11,393.3	11,392.4	0.64	11,393.3	11,393.9	0.77	11,393.3	11,394.6	0.62	11,352.3	11,353.3	0.93	11,393.3	11,392.9	0.59	11,393.3	11,392.8	0.66	11,393.3	11,393.2	0.62
YtxH domain-containing proteins	12,125.7	12,128.6	0.41	12,052.8	12,057.9	0.23	12,125.7	12,128.6	0.59	12,106.7	12,107.5	0.60	12,125.7	12,126.1	0.43	12,098.7	12,096.9	0.86	12,125.7	12,129.6	0.61
L20	13,138.4	13,139.1	0.13	13,099.4	13,100.2	0.03	13,138.4	13,139.3	0.08	13,110.4	13,110.5	0.89	13,138.4	13,138.9	0.16	13,138.4	13,139.1	0.14	13,138.4	13,139.0	0.11

^*a*^ Abbreviations: *m/z*, mass to charge ratio; NA, not assigned; SE, standard error.

To distinguish *Elizabethkingia* at the species level using MALDI-TOF MS, the combination of appropriate peaks of ribosomal L29, L30, S21, and YtxH domain-containing proteins was selected as a biomarker.

As shown in Fig. 2 and Table 1, ribosomal protein L29: The corresponding theoretical peaks were *m/z* 7,032.1 for *E. anophelis* NCTC 13869^T^, *E. bruuniana* CIP 111191^T^, *E. meningoseptica* NBRC 12535^T^, *E. miricola* JCM 11413^T^, and *E. ursingii* CIP 111192^T^; *m/z* 7,088.2 for *E. argenteiflava* JCM 32097^T^; and *m/z* 7,060.1 for *E. occulta* CIP 111193^T^ (Fig. 2; Table 1).

Ribosomal protein L30: The corresponding theoretical peaks were *m/z* 6,317.5 for *E. anophelis* NCTC 13869^T^; *m/z* 6,274.4 for *E. argenteiflava* JCM 32097^T^; *m/z* 6,333.5 for *E. bruuniana* CIP 111191^T^, *E. miricola* JCM 11413^T^, *E. ursingii* CIP 111192^T^, and *E. occulta* CIP 111193^T^; and *m/z* 6,283.5 for *E. meningoseptica* NBRC 12535^T^ (Fig. 2; Table 1).

Ribosomal protein S21: The corresponding theoretical peaks were *m/z* 7,643.0 for *E. anophelis* NCTC 13869^T^; *m/z* 7,647.0 for *E. argenteiflava* JCM 32097^T^; *m/z* 7,585.9 for *E. bruuniana* CIP 111191^T^, *E. miricola* JCM 11413^T^, *E. ursingii* CIP 111192^T^, and *E. occulta* CIP 111193^T^; and *m/z* 7,657.0 for *E. meningoseptica* NBRC 12535^T^ (Fig. 2; Table 1).

YtxH domain-containing proteins: The corresponding theoretical peaks were *m/z* 12,125.7 for *E. anophelis* NCTC 13869^T^, *E. bruuniana* CIP 111191^T^, *E. miricola* JCM 11413^T^, and *E. occulta* CIP 111193^T^; *m/z* 12,052.8 for *E. argenteiflava* JCM 32097^T^; *m/z* 12,106.7 for *E. meningoseptica* NBRC 12535^T^; and *m/z* 12,098.7 for *E. ursingii* CIP 111192^T^ (Fig. 2; Table 1).

Of these 14 biomarker peaks, the amino acid sequences of L29, L30, S21, and YtxH domain-containing proteins were unique among the *Elizabethkingia* species. Compared to the amino acid sequence of L29 in *E. anophelis*, there were four substitutions in the amino acid sequence of *E. argenteiflava*, one in *E. ursingii*, and one in *E. occulta* (Fig. S1A). Compared to the amino acid sequence of L30 in *E. anophelis*, there were four substitutions in *E. argenteiflava*, one in *E. bruuniana*, four in *E. meningoseptica*, one in *E. miricola*, one in *E. ursingii*, and one in *E. occulta* (Fig. S1B). Compared to the amino acid sequence of S21 in *E. anophelis*, there were seven substitutions in *E. argenteiflava*, one in *E. bruuniana*, one in *E. meningoseptica*, one in *E. miricola*, one in *E. ursingii*, and one in *E. occulta* (Fig. S1C). Compared to the amino acid sequence of YtxH domain-containing proteins of *E. anophelis*, there were 22 substitutions in *E. argenteiflava*, 9 in *E. meningoseptica*, and 1 in *E. ursingii* (Fig. S1D).

MALDI-TOF MS proteotyping was able to distinguish *E. anophelis*, *E. argenteiflava*, *E. bruuniana/E. miricola*, *E. meningoseptica*, *E. ursingii*, and *E. occulta* using the four biomarkers, including L29, L30, S21, and YtxH domain-containing proteins. However, *E. bruuniana* and *E. miricola* were not well separated by MALDI-TOF MS proteotyping.

### Assessment using clinical, environmental, and reference strains

The analyses of L29, L30, S21, and YtxH domain-containing proteins were evaluated using 29 clinical and environmental strains of *Elizabethkingia* species by MALDI-8020 (Table 2) and Microflex LT/SH (Table S3). The identification using Microflex LT/SH with the Biotyper database revealed that the strains of *E. anophelis*, *E. meningoseptica*, and *E. miricola* could identify correctly, but the other strains, including *E. argenteiflava*, *E. bruuniana*, *E. ursingii*, and *E. occulta*, could not identify at the species level (Table S3). Nine *E. anophelis* strains had identical peaks to the theoretical masses at *m/z* 7,032.1 for L29; *m/z* 6,317.5 for L30; *m/z* 7,643.0 for S21; and *m/z* 12,125.7 for YtxH domain-containing proteins. Three *E. bruuniana* and four *E. miricola* strains had identical peaks to the theoretical masses at *m/z* 7,032.1 for L29; *m/z* 6,333.5 for L30; *m/z* 7,585.9 for S21; and *m/z* 12,125.7 for YtxH domain-containing proteins. Three *E. meningoseptica* strains had identical peaks to the theoretical masses at *m/z* 7,032.1 for L29; *m/z* 6,283.5 for L30; *m/z* 7,657.0 for S21; and *m/z* 12,106.7 for YtxH domain-containing proteins. Two *E. ursingii* strains had identical peaks to the theoretical masses at *m/z* 7,032.1 for L29; *m/z* 6,333.5 for L30; *m/z* 7,585.9 for S21; and *m/z* 12,098.7 for YtxH domain-containing proteins. One *E. occulta* strain had identical peaks to the theoretical masses at *m/z* 7,060.1 for L29; *m/z* 6,333.5 for L30; *m/z* 7,585.9 for S21; and *m/z* 12,125.7 for YtxH domain-containing proteins.

**TABLE 2:** Average peaks of biomarker proteins in clinical and environmental strains of *Elizabethkingia* species by MALDI-8020

Species	Strains	Average of L29	SE	Average of L30	SE	Average of S21	SE	Average of YtxH domain-containing proteins	SE
*Elizabethkingia anophelis* ^*a*^	Type strain	7,032.6	0.12	6,318.2	0.19	7,643.0	0.08	12,128.6	0.41
	26380	7,032.0	0.20	6,318.3	0.35	7,641.9	0.09	12,125.8	0.94
	27288	7,032.4	0.06	6,318.3	0.08	7,642.9	0.06	12,128.3	0.25
	27513	7,031.5	0.64	6,316.9	0.71	7,642.1	0.58	12,128.6	0.39
	27521	7,032.1	0.14	6,317.6	0.23	7,642.7	0.14	12,128.6	0.33
	35834	7,032.7	0.18	6,318.5	0.24	7,643.0	0.09	12,127.1	0.70
	37341	7,032.4	0.12	6,318.2	0.14	7,642.8	0.10	12,127.9	0.32
	GS761	7,032.4	0.07	6,318.2	0.06	7,642.8	0.08	12,128.3	0.37
	GS777	7,032.4	0.10	6,318.0	0.11	7,642.9	0.09	12,127.3	0.35
	GS780	7,032.5	0.19	6,318.1	0.37	7,643.0	0.08	12,127.7	0.79
*Elizabethkingia argenteiflava* ^*b*^	type strain	7,088.5	0.03	6,275.3	0.05	7,647.0	0.05	12,057.9	0.23
*Elizabethkingia bruuniana* ^*c*^	type strain	7,032.1	0.40	6,333.4	0.51	7,585.7	0.32	12,128.6	0.59
	CCUG 69504	7,031.1	0.26	6,332.6	0.27	7,585.0	0.23	12,129.1	0.37
	CCUG 69513	7,031.9	0.15	6,334.1	0.11	7,585.7	0.10	12,128.3	0.71
	CCUG 69522	7,032.0	0.29	6,333.0	0.50	7,585.7	0.15	12,129.0	0.51
*Elizabethkingia meningoseptica* ^*d*^	type strain	7,032.5	0.15	6,284.1	0.31	7,657.0	0.08	12,107.5	0.60
	CCUG 69507	7,032.0	0.27	6,283.6	0.39	7,656.7	0.17	12,109.2	0.84
	CCUG 69515	7,032.5	0.22	6,284.3	0.27	7,656.9	0.14	12,108.3	0.31
	25547	7,032.4	0.24	6,284.2	0.38	7,657.0	0.13	12,110.7	0.23
*Elizabethkingia miricola* ^*c*^	type strain	7,032.4	0.13	6,334.0	0.20	7,585.9	0.23	12,126.1	0.43
	CCUG 69494	7,031.6	0.11	6,332.5	0.07	7,585.3	0.12	12,129.1	0.39
	CCUG 69519	7,032.1	0.35	6,333.7	0.54	7,585.4	0.16	12,128.6	0.77
	35660	7,032.2	0.21	6,332.8	0.41	7,585.6	0.08	12,127.8	0.56
	GS778	7,032.0	0.15	6,333.3	0.13	7,585.6	0.10	12,128.3	0.49
*Elizabethkingia ursingii* ^*e*^	type strain	7,032.8	0.18	6,334.6	0.32	7,586.0	0.06	12,096.9	0.86
	CCUG 69498	7,033.1	0.32	6,333.7	0.35	7,586.2	0.64	12,100.8	0.92
	CCUG 69517	7,033.0	0.21	6,333.7	0.22	7,585.5	0.15	12,098.4	0.19
*Elizabethkingia occulta* ^*f*^	type strain	7,060.3	0.08	6,333.6	0.21	7,586.0	0.03	12,129.6	0.61
	CCUG 69497	7,059.8	0.23	6,333.4	0.28	7,585.5	0.26	12,127.6	0.31

^*a*^ The theoretical masses of L29, L30, S21, and YtxH domain-containing proteins are *m/z* 7,032.1, *m/z* 6,317.5, *m/z* 7,643.0, and *m/z* 12,125.7, respectively.

^*b*^ The theoretical masses of L29, L30, S21, and YtxH domain-containing proteins are *m/z* 7,088.2, *m/z* 6,274.4, *m/z* 7,647.0, and *m/z* 12,052.8, respectively.

^*c*^ The theoretical masses of L29, L30, S21 and YtxH domain-containing proteins are *m/z* 7032.1, *m/z* 6333.5, *m/z* 7585.9 and *m/z* 12125.7, respectively.

^*d*^ The theoretical masses of L29, L30, S21, and YtxH domain-containing proteins are *m/z* 7,032.1, *m/z* 6,283.5, *m/z* 7,657.0, and *m/z* 12,106.7, respectively.

^*e*^ The theoretical masses of L29, L30, S21, and YtxH domain-containing proteins are *m/z* 7,032.1, *m/z* 6,333.5, *m/z* 7,585.9, and *m/z* 12,098.7, respectively.

^*f*^ The theoretical masses of L29, L30, S21, and YtxH domain-containing proteins are *m/z* 7,060.1, *m/z* 6,333.5, *m/z* 7,585.9, and *m/z* 12,125.7, respectively.

All 29 strains, including 9 *E. anophelis*, 3 *E. bruuniana*, 3 *E. meningoseptica*, 4 *E. miricola*, 2 *E. ursingii*, and 1 *E. occulta*, had identical peaks of the four biomarkers for each type strain.

A cluster analysis using the MALDI-TOF MS data revealed that *Elizabethkingia* species were classified into six groups, including *E. anophelis*, *E. argenteiflava*, *E. bruuniana/E. miricola*, *E. meningoseptica*, *E. ursingii*, and *E. occulta* (Fig. S2). It seems difficult to distinguish *E. bruuniana* and *E. miricola* strains based on the MALDI-TOF MS profiles.

## DISCUSSION

MALDI-TOF MS proteotyping is useful for both accurate and rapid identification of *Elizabethkingia* species. Conventional methods, such as API/ID32 Phenotyping Kits (bioMérieux), Phoenix 100 ID/AST Automated Microbiology System (Becton Dickinson), and Vitek 2 Automated Identification System (bioMérieux), require at least 4 h to identify *E. anophelis*, *E. meningoseptica*, and *E. miricola*. While Vitek MS (bioMérieux) and Bruker Biotyper MS (Bruker Daltonics) can identify only three species, including *E. anophelis*, *E. meningoseptica*, and *E. miricola*, because the database of the remaining four species is not included in the two MALDI. Our present study suggests that identification using 16S rRNA gene sequencing is difficult for distinguishing the seven *Elizabethkingia* species due to high sequence similarities. Lin *et al*. revealed that the 16S rRNA gene sequences of *E. bruuniana*, *E. miricola*, *E. occulta*, and *E. ursingii* strains exhibited the highest identity rate with other type strains and could not differentiate by the phylogenetic tree (20). Only WGS-based identification can distinguish *Elizabethkingia* species, but it is complicated and time-consuming. Predicting MALDI-TOF MS spectra using the GPMsDB will be useful for exploring of biomarker peaks. In this study, 14 detected peaks were assigned with annotated proteins, and four biomarker peaks, including ribosomal L29, L30, S21, and YtxH domain-containing proteins, were screened as biomarkers for detecting *Elizabethkingia* species.

The peaks likely representing L29, L30, S21, and YtxH domain-containing proteins are useful biomarkers for distinguishing *Elizabethkingia* at the species level. Cheng *et al.* reported that *m/z* 7,643.7 and *m/z* 10,320.9 are specific peaks for detecting *E. anophelis*; *m/z* 3,141.5 and *m/z* 12,109.1 for detecting *E. meningoseptica*; and *m/z* 3,792.5 and *m/z* 7,586.6 for detecting *E. miricola* and other species (21). In this study, *E. anophelis*, *E. argenteiflava*, *E. bruuniana/E. miricola, E. meningoseptica*, *E. ursingii*, and *E. occulta* could be distinguished using MALDI-TOF MS, but *E. bruuniana* and *E. miricola* could not be separated. Previous studies have reported that the phylogenic tree by 16S rRNA gene and *rpoB* gene sequencing between *E. bruuniana* and *E. miricola* are similar, leading to frequent misidentifications (20, 22).

**Fig 3:**
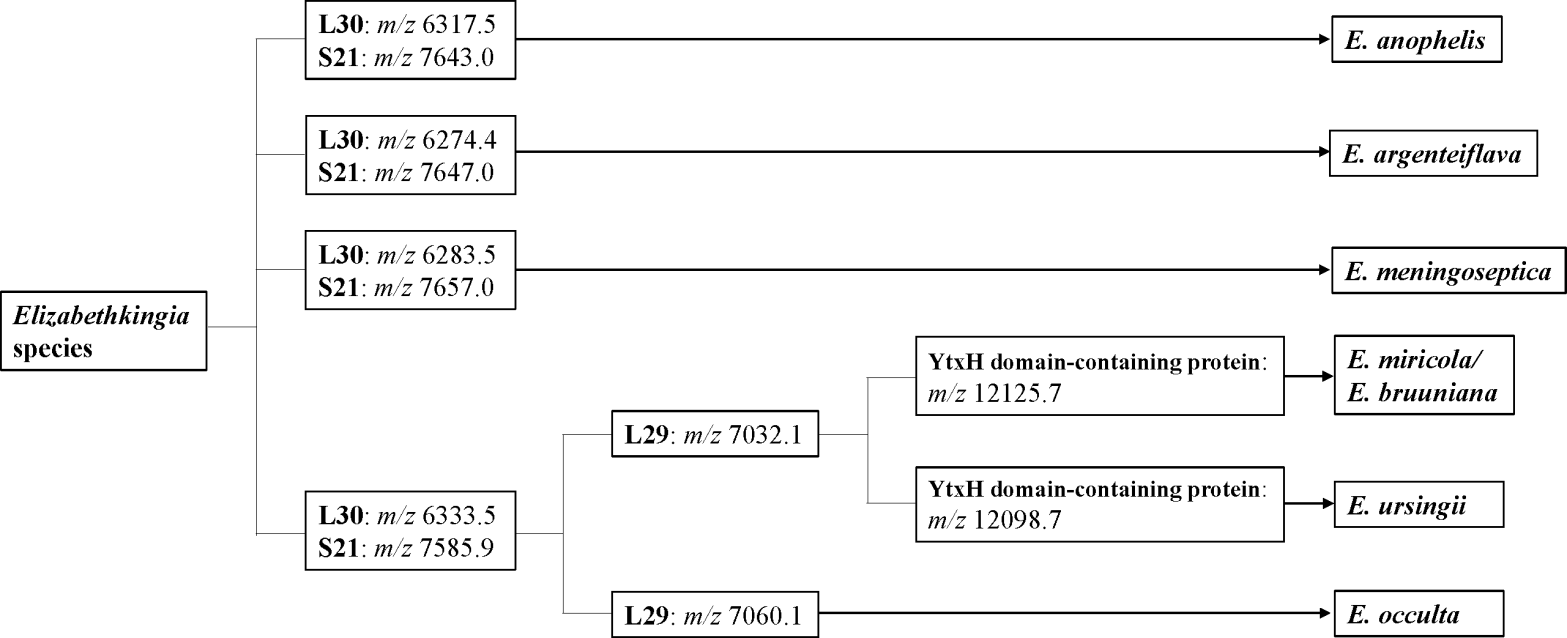
Workflow for the identification of phylotypes of *Elizabethkingia* species by MALDI-TOF MS proteotyping.

Figure 3 shows the workflow for the rapid identification of *Elizabethkingia* species by MALDI-TOF MS. Seven *Elizabethkingia* species are classified into six types: *E. anophelis*, *E. argenteiflava*, *E. bruuniana/E. miricola*, *E. meningoseptica*, *E. ursingii*, and *E. occulta*. This workflow, based on four biomarker peaks, will be useful for identifying *Elizabethkingia* species. In the future, it is necessary to update this workflow with continuous analysis using the GPMsDB, which assigns taxonomic identification of bacterial and archaeal cells with user-provided MALDI-TOF mass spectrometry profiles (15).

This study has a limitation: only a few clinical and environmental strains were obtained. It is necessary to confirm the findings using more diverse strains obtained from other hospital laboratories in different countries in the future.

In conclusion, the detection of the mass peaks of L29, L30, S21, and YtxH domain-containing proteins by MALDI-TOF MS proteotyping will be useful for the accurate and rapid descrimination of *Elizabethkingia* species.

## Data Availability

All data generated or analyzed during this study are included in this published article. The sequence data generated in this study have been submitted to the GenBank (DDBJ database; http://getentry.ddbj.nig.ac.jp/) under accession numbers SAMD00791561-SAMD00791568 and SAMD00787900-SAMD00787903.

## SUPPLEMENTAL MATERIAL

Figure S1 spectrum.02454-24-s0001.tiff Amino acid sequence alignment of ribosomal protein L29 (A), L30 (B), S21(C), and YtxH domain-containing proteins (D) of *Elizabethkingia* species.

Figure S2 spectrum.02454-24-s0002.tiff Cluster analysis of 29 clinical and environmental strains of *Elizabethkingia* species.

Table S1 spectrum.02454-24-s0003.xlsx NGS information.

Table S2 spectrum.02454-24-s0004.xlsx Comparison of *Elizabethkingia* type strains using ANI based on WGS and 16S rRNA sequence identities.

Table S3 spectrum.02454-24-s0005.xlsx Average peaks of biomarker proteins in clinical and environmental strains of Elizabethkingia species by Microflex LT/SH.

ASM does not own the copyrights to Supplemental Material that may be linked to, or accessed through, an article. The authors have granted ASM a non-exclusive, world-wide license to publish the Supplemental Material files. Please contact the corresponding author directly for reuse.
