# Supplementary figures and images for: Identification of Elizabethkingia species by MALDI-TOF MS proteotyping
Source: Microbiol Spectr. 2025 Feb 6;13(3):e02454-24. doi: 10.1128/spectrum.02454-24 (PMC11878014; doi:10.1128/spectrum.02454-24)

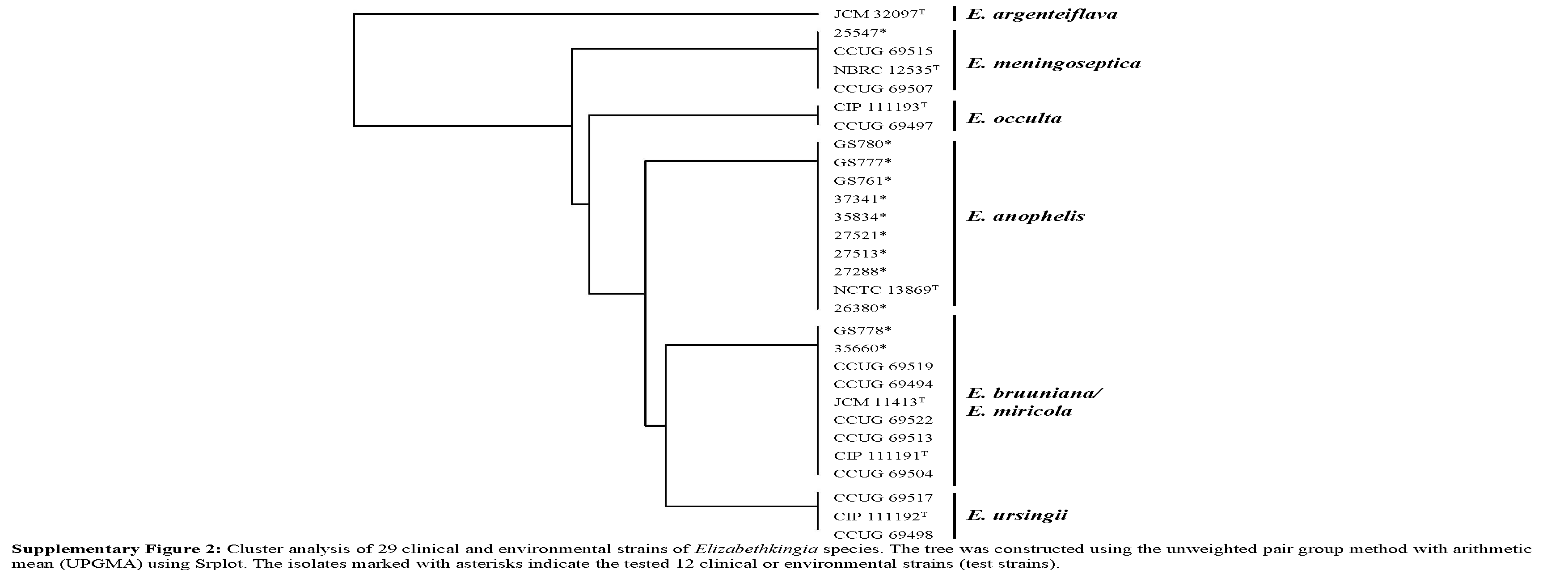

Supplement: Figure S2 — Cluster analysis of 29 clinical and environmental strains of Elizabethkingia species. [file spectrum.02454-24-s0002.tiff]
